# Exercise training improves cardiorespiratory fitness and oxygen uptake kinetics to a similar extent in early and late middle‐aged adults with type 2 diabetes

**DOI:** 10.14814/phy2.71107

**Published:** 2026-09-25

**Authors:** Catherine Kiely, Eamonn O'Connor, Donal O'Shea, Simon Green, Mikel Egaña

**Affiliations:** ^1^ School of Medicine, Department of Physiology Trinity College Dublin Dublin 2 Ireland; ^2^ Endocrinology, St Columcille's and St Vincent's Hospitals Dublin Ireland; ^3^ School of Health Sciences Western Sydney University Sydney Australia

**Keywords:** cardiac output, exercise intolerance, high‐intensity interval training, moderate‐intensity continuous training

## Abstract

To determine the exercise training–induced changes in peak oxygen uptake (V̇O_2peak_) and oxygen uptake kinetics in early and late middle‐aged adults with uncomplicated type 2 diabetes (T2D). Fifty inactive adults with T2D (16 females) were stratified into late middle‐aged (60–70 years) and early middle‐aged (37–59 years) cohorts and assigned to exercise training or control groups: late middle‐aged training (TR‐LMA, *n* = 12), late middle‐aged control (CON‐LMA, *n* = 9), early middle‐aged training (TR‐EMA, *n* = 17), or early middle‐aged control (CON‐EMA, *n* = 12). Training groups completed a supervised 12‐week aerobic and resistance programme. V̇O_2peak_ was assessed during incremental cycling while the time constant of the primary phase of oxygen uptake kinetics (τV̇O_2p_), blood pressure, and cardiac output (inert gas rebreathing) were measured during submaximal cycling before and after the intervention. V̇O_2peak_ increased and τV̇O_2_p decreased (*p* < 0.001) in both training groups: TR‐LMA (25.6 ± 5.7 to 28.2 ± 5.3 mL·kg^−1^·min^−1^; 39.8 ± 6.5 to 35.0 ± 7.3 s) and TR‐EMA (25.3 ± 5.3 to 28.1 ± 5.3 mL·kg^−1^·min^−1^; 43.3 ± 9.0 to 33.8 ± 8.2 s), with no between‐group differences. No changes occurred in CON‐LMA or CON‐EMA, and cardiovascular responses remained unchanged following training. Combined aerobic and resistance training similarly improved V̇O_2peak_ and *τ*V̇O_2p_ in early and late middle‐aged adults with T2D. The improvements in V̇O_2_ kinetics appear to occur independently of systemic cardiovascular adaptations.

## Graphical abstract

Supervised training improves V̇O_2peak_ and V̇O_2_ kinetics to a similar extent in early and late middle‐aged adults with type 2 diabetes, showing equivalent capacity for adaptation.

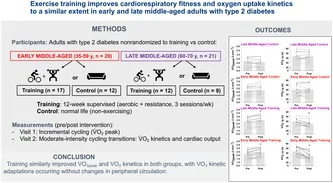

## 1 INTRODUCTION

The prevalence of type 2 diabetes (T2D) increases markedly with advancing age, contributing substantially to the growing global burden of the disease. As global populations continue to age, alongside persistent sedentary behavior and rising obesity rates, the clinical and economic burden of T2D is projected to escalate further. Preserving functional capacity and reducing cardiovascular risk across middle age and later life are therefore key clinical priorities in individuals with T2D. In this regard, exercise remains a cornerstone of T2D management because of its well‐established benefits for glycaemic control and cardiovascular risk reduction (Wei et al., 2000). However, individuals with T2D commonly exhibit impaired exercise capacity, which may limit their ability to meet physical activity recommendations (Johnson et al., 2005; Morrato et al., 2007). Specifically, lower peak oxygen uptake (V̇O_2peak_) is consistently observed across age groups in T2D compared with individuals without the condition (Alvares et al., 2024; Green et al., 2015; Mac Ananey et al., 2011; O'Connor et al., 2012, 2015; Regensteiner et al., 1998). Given that V̇O_2peak_ is a strong predictor of cardiovascular and all‐cause mortality (Wei et al., 2000), reductions in aerobic capacity carry important prognostic implications.

Beyond maximal capacity, T2D also alters submaximal exercise responses. Adults with T2D under 60 years demonstrate slower oxygen uptake (V̇O_2_) kinetics during moderate‐ and high‐intensity exercise, reflected by a prolonged time constant of the primary phase of the V̇O_2_ response (τV̇O_2p_) (Bauer et al., 2007; Gildea et al., 2021; Kiely et al., 2015; Mac Ananey et al., 2011; O'Connor et al., 2012; O'Connor et al., 2015), a key indicator of exercise tolerance (Hughson et al., 2001; Poole & Jones, 2012). Slower kinetics increase the oxygen deficit at exercise onset, promote greater reliance on substrate‐level phosphorylation and may contribute to earlier fatigue (Jones & Poole, 2005). Mechanisms influencing these impairments include reduced left ventricular filling (Wilson, Wilkins, et al., 2017; Wilson, Wilson, et al., 2017), impaired peripheral vasodilation and microvascular function (Bauer et al., 2007; Gildea et al., 2019; Gildea et al., 2021; Kiely et al., 2014; Mac Ananey et al., 2011; Poitras et al., 2015; Rocha et al., 2019; Wilkerson et al., 2011), and restricted muscular oxygen extraction (Kelley et al., 2002). Importantly, τV̇O_2p_ appears relatively preserved in adults with T2D in the seventh decade (60–70 years) compared with age‐matched controls, suggesting that age may modify the physiological phenotype of exercise impairment in T2D (O'Connor et al., 2015; Wilkerson et al., 2011). Whether this reflects age‐related ceiling effects (O'Connor et al., 2015) or compensatory cardiovascular and metabolic adaptations (Wilkerson et al., 2011) remains unclear.

Short‐term exercise interventions incorporating aerobic or combined aerobic and resistance training typically increase V̇O_2peak_ by approximately 15%–20% (Brandenburg et al., 1999; Gildea et al., 2021a; Macananey et al., 2012; O'Connor et al., 2025; Winding et al., 2018) and accelerate τV̇O_2p_ by 15%–30% in individuals with T2D (Brandenburg et al., 1999; Gildea et al., 2022; Gildea et al., 2021b; Green et al., 2020; Macananey et al., 2012). Such improvements are clinically meaningful, given the strong association between cardiorespiratory fitness and survival. However, most studies examining training‐induced adaptations in V̇O_2_ kinetics have involved younger or predominantly early middle‐aged cohorts (Brandenburg et al., 1999; Gildea et al., 2021b; Green et al., 2020; Macananey et al., 2012; O'Connor et al., 2025), with no direct comparisons across age groups. Consequently, it remains unclear whether late middle age/young older adults with T2D demonstrate V̇O_2peak_ and V̇O_2_ kinetics adaptations to structured exercise training that differ from those observed in early middle‐aged individuals with the disease, or whether age influences the magnitude and/or mechanisms of these responses.

Given the rising prevalence of T2D in older populations and the central role of exercise in disease management, clarifying age‐specific training responses has important implications for prescription and risk stratification. Accordingly, the present study investigated the effects of structured exercise training on V̇O_2peak_ and V̇O_2_ kinetics in early versus late middle‐aged adults with T2D. To further determine if changes in oxygen uptake kinetics were associated with central cardiovascular adaptations and enhanced oxygen delivery, cardiac output was assessed during submaximal exercise.

## 2 MATERIALS AND METHODS

### 2.1 Design and participants

This investigation formed one component of a larger project examining how sex‐related differences (Green et al., 2020) and treatment with pioglitazone (O'Connor et al., 2025) may influence the exercise training‐induced effects in V̇O_2peak_ and V̇O_2_ kinetics and cardiovascular responses during submaximal exercise in individuals with T2D. Fifty inactive adults with T2D were stratified by age into “late middle‐aged” (60–70 years; *n* = 21, 4 females) and “early middle‐aged” (37–59 years; *n* = 29, 12 females) cohorts. Two females were premenopausal. Inclusion required a confirmed diagnosis of T2D for under 10 years and HbA1c values below 10%. Exclusion criteria comprised insulin use, active smoking, contraindications to exercise testing or training, BMI above 40 kg/m^2^, and any major cardiovascular, hepatic, or renal disease (Table 1). Recruitment occurred through outpatient departments at St. Columcille's Hospital and St. Vincent's University Hospital in Dublin.

**TABLE 1:** Participants' clinical characteristics and activity levels at baseline and changes in physical characteristics and GXT variables before and after the 12‐week intervention in early and late middle‐aged participants with type 2 diabetes.

	Pre/Post intervention	CON‐LMA (*n* = 9)	CON‐EMA (*n* = 12)	TR‐LMA (*n* = 12)	TR‐EMA (*n* = 17)
Clinical characteristics and activity levels					
Sex (male, female), *n*		7, 2	7, 5	10, 2	10, 7
Age (years)		64 ± 3^a^	51 ± 3	63 ± 3^a^	54 ± 3
Time since diabetes diagnosis (yr)		5.2 ± 2.1	3.2 ± 1.8	4.5 ± 2.7	4.9 ± 3.5
Diabetes medication					
Diet only, *n* (%)		2 (22)	2 (17)	3 (25)	8 (47)
Metformin, *n* (%)		7 (78)	10 (83)	9 (75)	8 (47)
Sulfonylurea, *n* (%)		1 (11)	1 (8)	3 (25)	4 (24)
Glucagon‐like peptide‐1, *n* (%)		1 (11)	1 (8)		
Thiazolidinediones, *n* (%)		1 (11)	10 (83)		
Antihypertensive medication, *n* (%)		6 (67)	5 (42)	7 (58)	8 (47)
Statins, *n* (%)		7 (78)	5 (42)	8 (67)	8 (47)
Habitual physical activity					
LPA, h/day		4.4 ± 2.1	3.9 ± 2.3	4.3 ± 0.8	4.6 ± 2.0
MVPA, h/day		1.0 ± 0.6	0.8 ± 0.6	0.9 ± 0.6	1.1 ± 0.7
Changes in physical characteristics and GXT variables					
Body Mass (kg)	Pre	94.4 ± 11.9	89.5 ± 12.4	90.0 ± 12.6	86.9 ± 14.4
	Post	94.0 ± 12.4	89.8 ± 13.4	90.3 ± 12.1	86.7 ± 15.3
BMI (kg.m^−2^)	Pre	31.6 ± 3.5	31.7 ± 4.3	29.4 ± 2.4	31.6 ± 4.4
	Post	31.5 ± 3.6	31.9 ± 4.3	29.6 ± 2.4	31.5 ± 4.5
HbA_1c_ (%)	Pre	6.4 ± 0.7	6.3 ± 0.6	7.0 ± 1.1	6.7 ± 0.7
	Post	6.4 ± 0.7	6.4 ± 0.4	7.0 ± 1.2	6.6 ± 0.7
Glucose (mM)	Pre	7.3 ± 1.2	6.8 ± 1.3	7.9 ± 1.6	7.3 ± 1.1
	Post	7.8 ± 1.4	7.5 ± 2.1	8.2 ± 2.3	7.0 ± 1.2
V̇O_2peak_ (l.min^−1^)^c^, ^d^	Pre	2.19 ± 0.45	2.16 ± 0.56	2.31 ± 0.61	2.19 ± 0.51
	Post	2.11 ± 0.49	2.22 ± 0.67	2.57 ± 0.63^b^	2.43 ± 0.57^b^
V̇O_2peak_ (mL.min^−1^.kg^−1^)^c^, ^d^	Pre	23.4 ± 4.6	24.1 ± 5.4	25.6 ± 5.7	25.3 ± 5.3
	Post	22.7 ± 5.0	24.7 ± 6.3	28.2 ± 5.3^b^	28.1 ± 5.3^b^
PO_peak_ (W)^c^, ^d^	Pre	142 ± 27	134 ± 38	153 ± 47	136 ± 32
	Post	135 ± 30	138 ± 43	178 ± 51^b^	161 ± 40^b^
HR_peak_ (beat.min^−1^)	Pre	146 ± 15	159 ± 13	155 ± 17	153 ± 15
	Post	145 ± 13	163 ± 17	157 ± 20	149 ± 14
GET (W)^c^, ^d^	Pre	114 ± 25	111 ± 29	123 ± 34	115 ± 27
	Post	112 ± 27	116 ± 33	146 ± 40^b^	138 ± 35^b^
O_2_ pulse (mL.beats^−1^)^c^, ^d^	Pre	16.1 ± 3.2	15.3 ± 3.6	16.6 ± 3.1	16.8 ± 3.7
	Post	15.7 ± 3.7	15.3 ± 4.3	18.1 ± 2.7^b^	18.9 ± 3.5^b^

*Note*: Data are mean ± SD; *n* number of participants.

Abbreviations: BMI, body mass index; CON, non‐exercising control; EMA, early middle‐aged; GET, gas exchange threshold; HbA_1c_, glycated hemoglobin; HR, heart rate; LMA, late middle‐aged; LPA, light‐intensity physical activity; MVPA, moderate‐to‐vigorous physical activity; PO, power output; TR, exercise training; V̇O_2_, oxygen consumption.

^a^ Significantly different than both early middle‐aged groups (CON‐EMA and TR‐EMA) (*p* < 0.05).

^b^ Significantly different than pre‐training within the same group (*p* < 0.05).

^c^ Significant main effect of time (*p* < 0.05).

^d^ Significant time × group interaction (*p* < 0.05).

Participants first underwent a treadmill electrocardiogram stress assessment using the Bruce protocol and were non‐randomly assigned into one of four study groups: (1) late middle‐aged control (CON‐LMA: *n* = 9, 2 females, 64 ± 3 years), (2) early middle‐aged control (CON‐EMA: *n* = 12, 5 females, 51 ± 3 years); (3) late middle‐aged exercise training (TR‐LMA: *n* = 12, 2 females, 63 ± 3 years); and (4) early middle‐aged exercise training (TR‐EMA: *n* = 17, 7 females, 54 ± 3 years). Those in the exercise arms completed a 12‐week supervised training program, whereas control participants did not take part in any structured exercise during the study period. The non‐random allocation primarily reflected differences in participants' ability to travel to the training facility. All volunteers provided written informed consent. Ethical approval was granted by Trinity College Dublin (Ref: 131003) and St. Vincent's Healthcare. The study adhered to the guidelines set forth in the Declaration of Helsinki.

### 2.2 Exercise training intervention

The exercise intervention comprised up to 36 sessions over 12 weeks (three sessions per week). Adherence was defined as attendance at ≥32 sessions. All sessions were supervised by members of the research team and involved combined aerobic and resistance (concurrent) training, as this approach has been shown to produce greater reductions in HbA1c in individuals with T2D than either modality alone (Church et al., 2010). Further details of the training phases are provided elsewhere (O'Connor et al., 2025). Briefly, each session lasted 60–90 min and consisted of five components: (1) continuous cycling for 20–25 min at 70%–80% HR_max_; (2) upper and lower body flexibility exercises; (3) core strengthening exercises (sit‐ups, planks, and back extensions with 20–30 repetitions each); (4) resistance training for upper (bench press, upright row, shoulder press, lat pulldown) and lower body (leg extension, leg curl, leg press) at 60%–70% one‐repetition maximum for two sets of 10 reps; and (5) high‐intensity interval exercise consisting of up to three 90–120 s bouts at ~90% HR_max_ separated by two‐minute recovery periods. Training intensity and/or duration was progressively increased after weeks 4 and 8.

### 2.3 Testing

Participants attended the laboratory on two occasions both before and after the 12‐week intervention. All testing sessions were conducted at the same time of day and separated by 72 h. On the first visit, participants performed a graded exercise test (GXT) to volitional exhaustion on a cycle ergometer. The test started at 40 W and the workload increased by 30 W every 3 min until the required cadence could no longer be maintained (Egana et al., 2007). The gas exchange threshold (GET) was identified using the V‐slope method (Beaver et al., 1986), peak responses (including V̇O_2_) were defined as the highest 30‐s averaged values, and peak power output was calculated as previously described (Balmer et al., 2000).

During the second visit, participants completed six constant‐load cycling bouts. Each bout consisted of 3 min of ‘unloaded’ cycling (10 W) followed by 6 min at a workload corresponding to 80% of the individually determined GET. After the intervention, workloads were adjusted to reflect any change in power output at GET, ensuring the same relative intensity (80% GET) before and after training. Bouts were separated by 12‐min recovery periods to allow HR and blood lactate (measured in a subgroup, *n* = 25) to return to baseline. Oxygen uptake and HR were recorded continuously during the first four bouts, whereas CO and mean arterial pressure (MAP) were measured during the final two bouts. Previous work indicates that V̇O_2_ kinetic estimates are comparable whether repeated bouts are performed within a single day or across multiple days (Spencer et al., 2011). Venous blood samples were obtained after a 12‐h overnight fast 24–48 h prior to exercise testing. Participants were instructed to avoid caffeine and alcohol for 24 h before each laboratory visit.

### 2.4 Measurements

Pulmonary gas exchange was assessed breath‐by‐breath using a system (Innocor, Innovision A/S, Odense, Denmark) which measured expired air flow and respiratory gases sampled at the mouthport. This system computed V̇O_2_, rate of CO_2_ output (V̇CO_2_), minute ventilation (V̇_E_), and respiratory exchange ratio (RER). CO was measured using the same system but with the rebreathing of an inert gas mixture (sulfur hexafluoride and nitrous oxide). Cardiac output measurements were restricted to rest, 30 and 240 s of exercise because of the time required for adequate rebreathing and measurement (Jakovljevic et al., 2008; Mac Ananey et al., 2011). Heart rate was recorded every 5 s (S610i, Polar Electro Oy, Finland). Stroke volume (SV) was calculated from simultaneous HR and CO measurements. MAP was estimated from sphygmomanometric measurements of brachial arterial pressures (0.33 systolic + 0.66 diastolic) performed at the same times CO was measured. Systemic vascular conductance (SVC) was calculated from CO and MAP measurements (SVC = CO/MAP). All cardiovascular measurements were mean values of responses during two exercise bouts.

### 2.5 Analysis of kinetic responses of V̇O_2_ and cardiovascular variables

Analysis of V̇O_2_ kinetics was performed using data from the first four submaximal cycling bouts completed at a relative intensity of 80% GET before and after the intervention. Baseline V̇O_2_ was defined as the mean value during the final 60 s of unloaded cycling preceding the transition in workload. Breath‐by‐breath data from 0 to 240 s following the transition were interpolated to 1‐s intervals, temporally aligned, averaged across bouts, and smoothed with a five‐second moving average (Keir et al., 2014). The processed responses were fitted using either a monoexponential (Equation 1) or biexponential (Equation 2) model according to the response profile. Most responses (83%) were adequately described by a monoexponential function representing the primary adjustment phase. When a distinct slow component was present (17% of cases; mean ± SD amplitude: 110 ± 73 mL·min^−1^), a biexponential model was applied. The equations are:

(1) $$\dot{V}O_{2}\;\left(t\right)=a+A_{1}.\left(1-e^{–\left(\left(t–\text{TD1}\right)/\tau 1\right)}\right).F_{1}$$

(2) $$\dot{V}O_{2}\;\left(t\right)=a+A_{1}.\left(1-e^{–\left(\left(t–\text{TD1}\right)/\tau 1\right)}\right).F_{1}+A_{2}.\left(1-e^{–\left(\left(t–\text{TD2}\right)/\tau 2\right)}\right).F_{2}$$

In the model equations, *a* represents baseline V̇O_2_ at 10 W; A₁ and A_2_ denote the amplitudes of the primary and slow phases; *τ*₁ and *τ*_2_ their respective time constants; and TD₁ and TD_2_ the associated time delays. Conditional terms F₁ and F_2_ ensure each phase is fitted only after its delay. To minimize the influence of the cardiodynamic phase, the first 20 s of data were excluded, although TD₁ was allowed to vary during fitting to improve parameter estimation. Because T2D primarily affects the primary phase, only *τ*₁ (*τ*V̇O_2p_) and derived variables are reported. Model fitting used a two‐step weighted least‐squares non‐linear regression procedure with removal of outliers after the initial step. V̇O_2_ gain (A₁/Δpower) and end‐exercise V̇O_2_ (mean of the final 30 s) were also calculated.

Dynamic responses of CO, SV, MAP, SVC, and HR were derived from the averaged responses during the final two exercise bouts. Dynamic responses were quantified by expressing the change observed during the first 30 s of exercise as a percentage of the total change measured over 240 s (Δ30/Δ240 × 100%).

### 2.6 Statistical analyses

Age, duration of diabetes, physical activity levels, baseline physical characteristics, and GXT‐derived variables were initially compared between groups using one‐way ANOVA. Changes in physical characteristics, peak physiological responses, V̇O_2_ kinetic parameters, and cardiovascular dynamic variables were analyzed using a two‐factor mixed ANOVA with factors for time (pre‐training vs. post‐training) and group (CON‐LMA, CON‐EMA, TR‐LMA, TR‐EMA). When significant effects were detected, Bonferroni‐adjusted post hoc tests were used to identify differences between groups. Differences in the magnitude of training‐induced changes between the exercise groups were further examined using independent *t*‐tests. Statistical significance was accepted at *p* < 0.05. All data are presented as mean ± SD.

## 3 RESULTS

### 3.1 Participant characteristics and GXT variables

**FIGURE 1:**
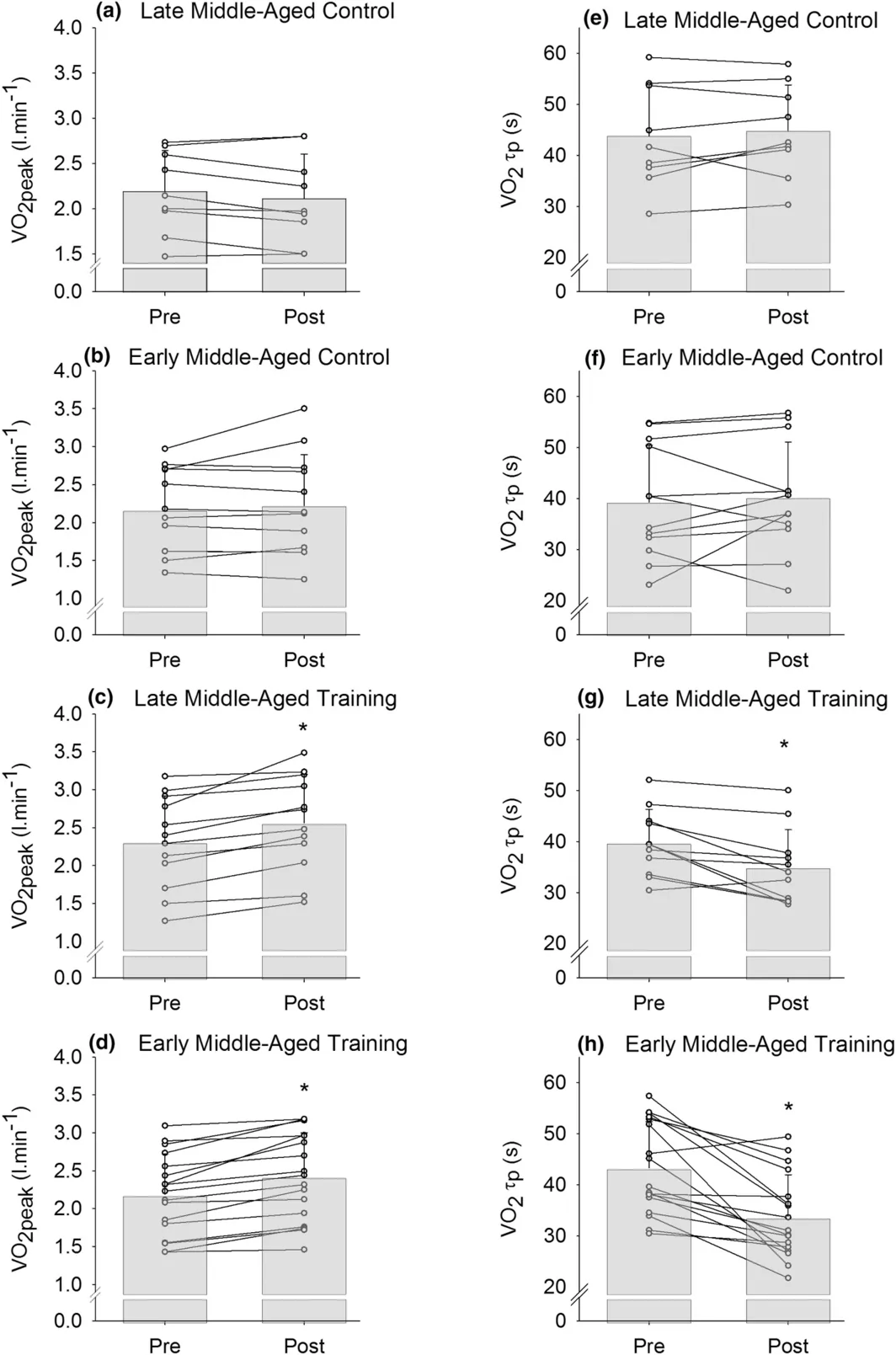
Individual and mean responses of peak oxygen uptake (V̇O_2peak_, panels A–D) and time constant of the primary V̇O_2_ response (τV̇O_2p_, panels E–H) in the four experimental groups (Late middle‐aged control, *n* = 9; Early middle‐aged control, *n* = 12; Late middle‐aged training, *n* = 12; Early middle‐aged training, *n* = 17) before and after the 12‐week intervention. Error bars represent SD. A two‐factor mixed ANOVA was used to carry out the analyses. * Significantly different than pre‐training within the same group (*p* < 0.05).

Participant characteristics and responses from the GXT are presented in Table 1, and individual and mean group changes in V̇O_2peak_ are illustrated in Figure 1. Adherence to the exercise programme was high in both training groups (TR‐LMA, TR‐EMA), with participants completing an average of 35 sessions (range: 32–36) out of a possible 36. Participants in both early middle‐aged groups were younger than in the late middle‐aged groups (*p* < 0.001). Duration of diabetes and habitual physical activity levels were similar across groups (all *p* > 0.29). Baseline physical characteristics and GXT‐derived variables were also not different among groups (all *p* > 0.06). Body mass and HbA1c remained unchanged in all groups following the 12‐week intervention. In contrast, significant increases were observed in absolute and relative V̇O_2peak_, peak power output (PO_peak_), O_2_ pulse, and power output at GET in both exercise groups after training (for all variables, main effect of time, *p* < 0.001 and significant time × group interaction, *p* < 0.001). The magnitude of these improvements, expressed both in absolute terms and as percentages, did not differ between TR‐LMA and TR‐EMA (*t*‐tests, all *p* > 0.2).

### 3.2 VO_2_ kinetics

Estimates of the V̇O_2_ kinetic parameters and derived measurements for the primary phase are summarized in Table 2 together with the statistical results, while individuals and mean changes of the time constant of the primary V̇O_2_ response (τV̇O_2p_) are shown in Figure 1. Following the intervention, τV̇O_2p_ was reduced, indicating faster kinetics in both exercise groups only (significant time × group interaction, *p* < 0.001). The % magnitude of this improvement, although numerically larger in TR‐EMA compared with TR‐LMA (~21% vs. ~12% respectively), was not significantly different between the groups (*t*‐tests, *p* = 0.11). Significant main effects of time were also observed for the primary amplitude of V̇O_2_ and end‐exercise V̇O_2_ (*p* = 0.005 and *p* = 0.003, respectively). Although these increases were mainly observed in the exercise groups, the time × group interactions were not statistically significant (*p* = 0.20 and *p* = 0.14).

**TABLE 2:** V̇O_2_ kinetics parameters and changes in cardiovascular variables during submaximal exercise before and after a 12‐week intervention in early and late middle‐aged male and female participants with type 2 diabetes.

	Pre/Post intervention	CON‐LMA (*n* = 9)	CON‐EMA (*n* = 12)	TR‐LMA (*n* = 12)	TR‐EMA (*n* = 17)
*V̇O*_*2*_ *kinetics parameters*					
V̇O_2_ base (l.min^−1^)	Pre	0.70 ± 0.10	0.72 ± 0.19	0.81 ± 0.13	0.68 ± 0.19
	Post	0.72 ± 0.11	0.72 ± 0.17	0.83 ± 0.18	0.73 ± 0.17
τV̇O_2p_ (s)^b^, ^c^	Pre	43.8 ± 10.1	39.3 ± 11.2	39.8 ± 6.5	43.3 ± 9.0
	Post	44.8 ± 9.0	40.2 ± 10.9	35.0 ± 7.3^a^	33.8 ± 8.2^a^
V̇O_2_ TD (s)	Pre	16.6 ± 8.6	14.8 ± 7.8	20.3 ± 9.3	15.6 ± 8.0
	Post	15.9 ± 9.5	15.1 ± 6.5	19.3 ± 6.8	18.9 ± 5.3
V̇O_2_ Amp (l.min^−1^)^b^	Pre	0.82 ± 0.28	0.82 ± 0.26	0.96 ± 0.34	0.88 ± 0.27
	Post	0.85 ± 0.16	0.83 ± 0.31	1.08 ± 0.35	1.02 ± 0.29
V̇O_2_ Gain (mL.min^−1^.W^−1^)	Pre	10.0 ± 1.7	10.4 ± 1.2	11.3 ± 3.4	10.8 ± 2.0
	Post	11.1 ± 1.4	10.0 ± 1.4	10.3 ± 1.2	10.2 ± 1.3
End V̇O_2_ (l.min^−1^)^b^	Pre	1.56 ± 0.30	1.56 ± 0.40	1.79 ± 0.40	1.56 ± 0.39
	Post	1.59 ± 0.24	1.61 ± 0.48	1.95 ± 0.48	1.79 ± 0.35
*Dynamic changes in cardiovascular variables*					
ΔCO_30_/ΔCO_240_ (%)	Pre	67 ± 15	71 ± 15	72 ± 12	64 ± 14
	Post	72 ± 12	69 ± 18	73 ± 8	69 ± 11
ΔHR_30_/ΔHR_240_ (%)	Pre	53 ± 10	48 ± 15	50 ± 18	50 ± 18
	Post	48 ± 14	51 ± 25	50 ± 8	52 ± 11
ΔSV_30_/ΔSV_240_ (%)	Pre	99 ± 46	105 ± 31	97 ± 32	90 ± 27
	Post	117 ± 36	99 ± 34	89 ± 33	103 ± 32
ΔMAP_30_/ΔMAP_240_ (%)	Pre	62 ± 29	65 ± 12	68 ± 15	72 ± 23
	Post	73 ± 18	78 ± 13	70 ± 19	67 ± 13
ΔSVC_30_/ΔSVC_240_ (%)	Pre	70 ± 30	79 ± 21	77 ± 12	66 ± 24
	Post	76 ± 16	71 ± 22	82 ± 23	72 ± 14

*Note*: Data are mean ± SD. Cardiovascular responses over 30 s are expressed as percentages of responses over 240 s of exercise (i.e., change from rest) to provide estimates of the dynamic responses of these variables during exercise.

Abbreviations: Amp, amplitude; base, baseline; CO, cardiac output; CON, non‐exercising control; EMA, early middle‐aged; End; end‐exercise; GET, gas exchange threshold; HR, heart rate; LMA, late middle‐aged; MAP, mean arterial pressure; SV, stroke volume; SVC, systemic vascular conductance; TD, time delay; TR, exercise training; τpV̇O_2_, time constant of V̇O_2_ during the primary phase.

^a^ Significantly different than pre‐training within the same group (*p* < 0.05).

^b^ Significant main effect of time (*p* < 0.05).

^c^ Significant time × group interaction (*p* < 0.05).

### 3.3 Cardiovascular dynamics

**FIGURE 2:**
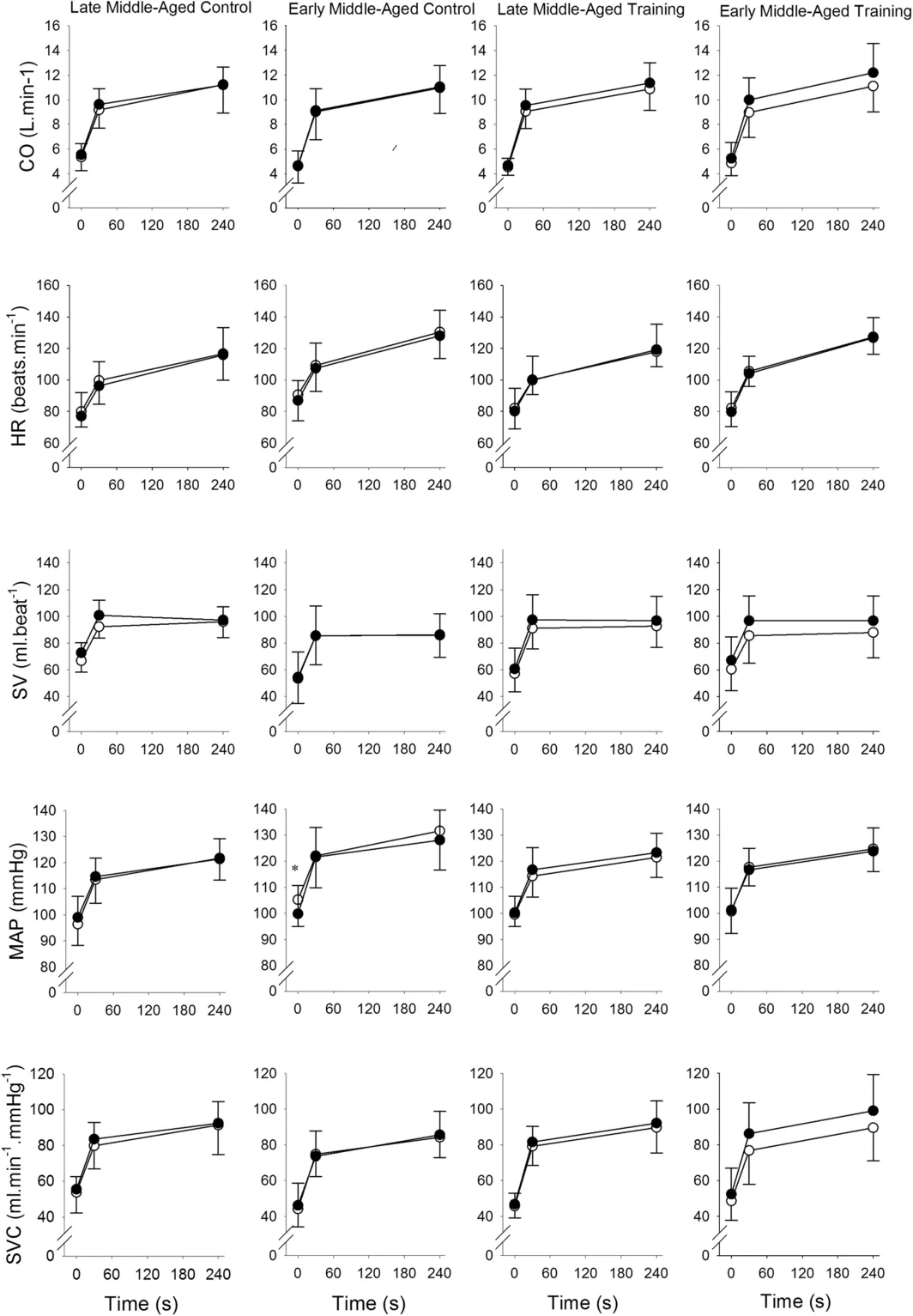
Mean ± SD cardiovascular responses at rest, 30 and 240 s of exercise before (open circles) and after (closed circles) the 12‐week intervention in the four experimental groups (Late middle‐aged control, *n* = 9; Early middle‐aged control, *n* = 12; Late middle‐aged training, *n* = 12; Early middle‐aged training, *n* = 17). Statistical outcomes are further described within the results section. CO, cardiac output; HR, heart rate; SV, stroke volume; MAP, mean arterial pressure; SVC, systemic vascular conductance. * Significantly different than pre‐training within the same group (*p* < 0.05).

Group mean cardiovascular responses are illustrated in Figure 2. Neither the exercise intervention nor the control period significantly altered the rate of adjustment of any cardiovascular variable (Table 2). However, significant main effects of time were observed for CO, SV, and SVC at 30 s and also for SV and SVC at 240 s (all *p* < 0.05), largely reflecting the higher exercise workloads achieved after training in the exercise groups. No significant time × group interactions were detected (all *p* > 0.06). At baseline, there was a main effect of time for SV (*p* < 0.05) while mean arterial pressure (MAP) was lower (*p* = 0.03) post‐intervention in the CON‐EMA group (group × time interaction, *p* = 0.045).

## 4 DISCUSSION

To our knowledge, this is the first study to examine exercise training‐induced changes in cardiorespiratory fitness and oxygen uptake kinetics across two middle‐aged cohorts of individuals with T2D and mild obesity.

The main findings were: (1) both exercise groups (TR‐LMA and TR‐EMA) showed significant improvements in V̇O_2peak_ and τV̇O_2p_, with no differences in the magnitude of adaptation between groups; and (2) the improvement in V̇O_2_ kinetics was not accompanied by alterations in cardiovascular dynamics. Taken together, these findings suggest that exercise training enhances both maximal and submaximal exercise tolerance to a magnitude not different between previously untrained “early middle‐aged” (40s–50s) and “late middle‐aged” (60s) individuals with T2D of similar disease duration and baseline physiological profile, and that the observed improvements in submaximal responses appear to occur independently of systemic cardiovascular adaptations.

### 4.1 Peak cardiorespiratory fitness

Both exercise groups exhibited clinically meaningful increases in V̇O_2peak_ (~12%). Given that V̇O_2peak_ is a strong independent predictor of cardiovascular and all‐cause mortality (Ross et al., 2016), such improvements are likely to translate into meaningful reductions in risk, particularly in late middle‐aged adults with T2D who exhibit elevated baseline cardiometabolic burden. The concomitant rise in O_2_ pulse, improved V̇O_2peak_ without a change in HR_max_, supports the presence of true physiological adaptation rather than differences in effort or test familiarization.

Mechanistically, exercise‐induced improvements in V̇O_2peak_ in individuals with T2D, relative to non‐exercising controls, have been attributed to both central and peripheral adaptations. Central adaptations may include increased left ventricular end‐diastolic volume and stroke volume, mediated by improved ventricular compliance and preload (Wilson et al., 2019). Peripheral mechanisms likely include enhanced microvascular oxygen delivery (Gildea et al., 2021b), improved vascular function (Schreuder et al., 2015), structural vascular remodeling (Madsen et al., 2015), and an expanded capillary‐to‐myocyte interface (Mortensen et al., 2019), potentially mediated by an increase in the number of red blood cell–perfused capillaries within skeletal muscle (Padilla et al., 2006).

Our laboratory previously showed that peak V̇O_2_ was reduced in participants with T2D aged <60 years (by ~22%) and in those aged 60–70 years (by ~12%) compared with their age‐matched healthy counterparts (O'Connor et al., 2015). The current study, which included participants with comparable disease duration and clinical characteristics, extends these observations by demonstrating that impairments in V̇O_2peak_ remain modifiable in these age groups with uncomplicated T2D.

### 4.2 Oxygen uptake kinetics during moderate‐intensity exercise

In addition to improvements in maximal capacity, both groups exhibited significant acceleration of V̇O_2_ kinetics during moderate‐intensity exercise (~12–21% reduction in *τ*V̇O_2p_). Given that *τ*V̇O_2p_ reflects the efficiency of the transition from rest to activity, these findings have clinical relevance. Faster kinetics reduce the oxygen deficit at exercise onset, limit reliance on substrate‐level phosphorylation, and may delay fatigue, thereby improving tolerance to everyday physical tasks, an effect particularly relevant for adults in late middle age who may be at increased risk of functional decline.

Although not directly established in T2D, evidence from other clinical populations, such as heart failure and chronic obstructive pulmonary disease, suggests that training‐induced changes in *τ*V̇O_2p_ are likely mediated by alterations in the rate of oxygen delivery to, and/or utilization by, the contracting skeletal muscle (Hughson, 2009; Poole & Jones, 2012). To examine potential central contributions, we assessed early (30 s) versus later (240 s) changes in cardiovascular variables during submaximal exercise. Consistent with recent findings (O‘Connor et al., 2025; Green et al., 2020), no significant alterations in systemic cardiovascular dynamics were detected following training, suggesting that large changes in central haemodynamic kinetics were unlikely to explain the observed acceleration in τV̇O_2p_.

Our previous baseline study suggested that slowed τV̇O_2p_ in middle‐aged males with T2D was at least partly attributable to blunted early SVC (estimated from the slope MAP and CO) responses and impaired muscle perfusion (O'Connor et al., 2015). The exaggerated pressor response during submaximal exercise in middle‐aged T2D participants, despite smaller rises in CO, implied reduced vascular conductance in contracting skeletal muscle. In contrast, older males with T2D did not exhibit this additional vascular impairment, consistent with the absence of further slowing in *τ*V̇O_2p_ beyond that attributable to aging alone (O'Connor et al., 2015). Others also reported unaltered *τ*V̇O_2p_ in older males with T2D compared with controls alongside a greater increase in muscle deoxygenation per unit of V̇O_2_, suggesting enhanced O_2_ extraction during exercise as a compensatory response to impaired muscle blood flow (Wilkerson et al., 2011). The present findings extend these observations by demonstrating that, despite differences in baseline exercise responses reported across age groups, structured exercise training improves dynamic oxygen uptake in both early and late middle‐aged adults with T2D without altering early SCV responses. This suggests that the training‐induced acceleration of V̇O_2_ kinetics is more likely driven by enhanced peripheral oxygen delivery–utilization matching rather than improvements in central oxygen delivery although peripheral adaptations were not measured in the current investigation. Such peripheral adaptations may include improved local matching of oxygen delivery to muscle metabolic demand (Gildea et al., 2021b) enhanced vasodilatory capacity, and improved dynamic microvascular responses (Green et al., 2022).

### 4.3 Clinical implications

Despite the increasing prevalence of T2D across middle and later life and elevated cardiovascular risk associated with the condition, responsiveness to exercise training, reflected in improvements in both maximal and submaximal exercise capacity, appears to be maintained across early and late middle age, with no clear evidence of reduced physiological plasticity. Overall, the data support the use of structured aerobic or combined training programmes as an effective approach to improve key functional physiological markers in this population.

### 4.4 Limitations

This study has several limitations that should be acknowledged. First, due to the limited temporal resolution of the inert gas rebreathing technique, cardiac output (CO) was not assessed continuously; therefore, subtle alterations in dynamic central haemodynamics cannot be excluded. Future studies employing higher‐resolution methodologies may help to further delineate the relative contributions of central and peripheral adaptations. Second, the proportion of males and females differed between groups. However, recent evidence indicates that adaptations in V̇O_2peak_ and V̇O_2_ kinetics following exercise training are not influenced by sex in individuals with type 2 diabetes (Green et al., 2020), supporting the inclusion of both sexes in the present study. Furthermore, excluding female participants from the analysis did not alter the main outcomes (data not shown). Nevertheless, the unequal sex distribution may have influenced baseline physiological characteristics, so future studies specifically investigating the age‐related differences in exercise adaptations should aim to recruit more balanced sex distributions across groups. Third, the proportion of participants managed with diet alone was numerically higher in the early middle‐aged training group, which may have influenced the study outcomes. Fourth, participants had relatively well‐controlled diabetes (mean HbA1c approximately 6.3%–7.0%), which may limit the generalisability of our findings to individuals with poorer glycaemic control or more advanced disease, who typically exhibit greater impairments in cardiorespiratory fitness and exercise capacity (Alvares et al., 2024; Soares & Lessard, 2024). Fifth, a concurrent training programme was chosen because of its high ecological validity in type 2 diabetes. However, the present study cannot determine the relative contributions of the aerobic and resistance components to the observed improvements in V̇O_2_peak and *τ*V̇O_2_p. Although the aerobic component was based on a previously published protocol that elicited improvements in systemic cardiovascular dynamics in adults with type 2 diabetes, no such changes were observed in the present study. Therefore, it cannot be excluded that the aerobic training stimulus was insufficient to elicit detectable central cardiovascular adaptations. Finally, the mean age difference between the early and late middle‐aged groups was relatively modest (~10 years). This limited age separation restricts the extent to which the present findings can be interpreted as evidence of age‐related differences in exercise trainability and limits their generalisability across a broader age range. Nevertheless, the present study provides a relevant benchmark for the expected magnitude of exercise‐training adaptations in individuals with T2D in their 40s–50s and 60s, despite previous evidence of impaired V̇O_2_ kinetics in those in their 40s–50s, but not in those in their 60s, relative to their respective age‐matched non‐diabetic controls (O'Connor et al., 2015; Wilkerson et al., 2011).

## 5 CONCLUSION

In conclusion, 12 weeks of structured exercise training significantly improved V̇O_2peak_ and accelerated V̇O_2_ kinetics in early and late middle‐aged adults with uncomplicated T2D and same disease duration. These adaptations were similar between age groups and were not accompanied by changes in systemic cardiovascular dynamics. The findings reinforce the clinical value of exercise training across early and late middle age in adults with T2D and highlight preserved physiological adaptability within these age groups. Further investigation is needed to determine whether comparable benefits extend to individuals at more advanced ages, including those over 70 years and/or with longer disease duration.

## AUTHOR CONTRIBUTIONS

**Catherine Kiely:** Conceptualization; formal analysis; investigation; methodology. **Eamonn O'Connor:** Conceptualization; formal analysis; investigation; methodology. **Donal O'Shea:** Conceptualization; funding acquisition; investigation; methodology; resources. **Simon Green:** Conceptualization; funding acquisition; investigation. **Mikel Egaña:** Conceptualization; formal analysis; funding acquisition; methodology; project administration; supervision.

## FUNDING INFORMATION

This research was supported by Research Ireland (Grant No. 08/RFP/BMT1342).

## CONFLICT OF INTEREST STATEMENT

The authors declare no conflicts of interest.

## Data Availability

Data will be made available on request within the limitations of the informed consent. Requests should be made to the corresponding author.

## References

[phy271107-bib-0001] Alvares, T. S. , Soares, R. N. , & Lessard, S. J. (2024). Cardiorespiratory fitness is impaired in type 1 and type 2 diabetes: A systematic review, meta‐analysis, and meta‐regression. Medicine and Science in Sports and Exercise, 56(9), 1553–1562. 10.1249/mss.0000000000003451 PMC11327000 38650120

[phy271107-bib-0002] Balmer, J. , Davison, R. C. , & Bird, S. R. (2000). Peak power predicts performance power during an outdoor 16.1‐km cycling time trial. Medicine and Science in Sports and Exercise, 32, 1485–1490. 10.1097/00005768-200008000-00018 10949016

[phy271107-bib-0003] Bauer, T. A. , Reusch, J. E. B. , Levi, M. , & Regensteiner, J. G. (2007). Skeletal muscle deoxygenation after the onset of moderate exercise suggests slowed microvascular blood flow kinetics in type 2 diabetes. Diabetes Care, 30(11), 2880–2885. 10.2337/dc07-0843 17675540

[phy271107-bib-0004] Beaver, W. L. , Wasserman, K. , & Whipp, B. J. (1986). A new method for detecting anaerobic threshold by gas exchange. Journal of Applied Physiology, 60, 2020–2027. 10.1152/jappl.1986.60.6.2020 3087938

[phy271107-bib-0005] Brandenburg, S. L. , Reusch, J. E. , Bauer, T. A. , Jeffers, B. W. , Hiatt, W. R. , & Regensteiner, J. G. (1999). Effects of exercise training on oxygen uptake kinetic responses in women with type 2 diabetes. Diabetes Care, 22(10), 1640–1646. 10.2337/diacare.22.10.1640 10526728

[phy271107-bib-0006] Church, T. S. , Blair, S. N. , Cocreham, S. , Johannsen, N. , Johnson, W. , Kramer, K. , Mikus, C. R. , Myers, V. , Nauta, M. , Rodarte, R. Q. , Sparks, L. , Thompson, A. , & Earnest, C. P. (2010). Effects of aerobic and resistance training on hemoglobin A1c levels in patients with type 2 diabetes: A randomized controlled trial. Journal of the American Medical Association, 304(20), 2253–2262. 10.1001/jama.2010.1710 PMC3174102 21098771

[phy271107-bib-0007] Egana, M. , Smith, S. , & Green, S. (2007). Revisiting the effect of posture on high‐intensity constant‐load cycling performance in men and women. European Journal of Applied Physiology, 99(5), 495–501. 10.1007/s00421-006-0365-8 17206442

[phy271107-bib-0008] Gildea, N. , McDermott, A. , Rocha, J. , Crognale, D. , Nevin, A. , O'Shea, D. , Green, S. , & Egaña, M. (2022). Low‐volume HIIT and MICT speed V̇O(2) kinetics during high‐intensity “work‐to‐work” cycling with a similar time‐course in type 2 diabetes. Journal of Applied Physiology, 133(2), 273–287. 10.1152/japplphysiol.00148.2022 35678744

[phy271107-bib-0009] Gildea, N. , McDermott, A. , Rocha, J. , O'Shea, D. , Green, S. , & Egaña, M. (2021a). Time course of changes in V̇o(2peak) and O(2) extraction during ramp cycle exercise following HIIT versus moderate‐intensity continuous training in type 2 diabetes. American Journal of Physiology. Regulatory, Integrative and Comparative Physiology, 320(5), R683–R696. 10.1152/ajpregu.00318.2020 33624548

[phy271107-bib-0010] Gildea, N. , McDermott, A. , Rocha, J. , O'Shea, D. , Green, S. , & Egaña, M. (2021b). Time‐course of V̇o(2) kinetics responses during moderate‐intensity exercise subsequent to HIIT versus moderate‐intensity continuous training in type 2 diabetes. Journal of Applied Physiology, 130(6), 1646–1659. 10.1152/japplphysiol.00952.2020 33792400

[phy271107-bib-0011] Gildea, N. , Rocha, J. , McDermott, A. , O'Shea, D. , Green, S. , & Egaña, M. (2019). Influence of type 2 diabetes on muscle deoxygenation during ramp incremental cycle exercise. Respiratory Physiology & Neurobiology, 269, 103258. 10.1016/j.resp.2019.103258 31349019

[phy271107-bib-0012] Gildea, N. , Rocha, J. , O'Shea, D. , Green, S. , & Egaña, M. (2021). Priming exercise accelerates pulmonary oxygen uptake kinetics during “work‐to‐work” cycle exercise in middle‐aged individuals with type 2 diabetes. European Journal of Applied Physiology, 121(2), 409–423. 10.1007/s00421-020-04518-y 33084929

[phy271107-bib-0013] Green, S. , Egaña, M. , Baldi, J. C. , Lamberts, R. , & Regensteiner, J. G. (2015). Cardiovascular control during exercise in type 2 diabetes mellitus. Journal Diabetes Research, 2015, 654204. 10.1155/2015/654204 PMC4396731 25918732

[phy271107-bib-0500] Green, S. , Kiely, C. , O‘Connor, E. , Gildea, N. , O‘Shea, D. , & Egaña, M. (2020). Effects of exercise training and sex on dynamic responses of O_2_ uptake in type 2 diabetes. Applied Physiology, Nutrition, and Metabolism, 45(8), 865–874. 10.1139/apnm-2019-0636 32134683

[phy271107-bib-0014] Green, S. , Kiely, C. , O'Connor, E. , Gildea, N. , O'Shea, D. , & Egaña, M. (2022). Differential effects of sex on adaptive responses of skeletal muscle vasodilation to exercise training in type 2 diabetes. J Diabetes Complications, 36(1), 108098. 10.1016/j.jdiacomp.2021.108098 34887186

[phy271107-bib-0015] Hughson, R. L. (2009). Oxygen uptake kinetics: Historical perspective and future directions. Applied Physiology, Nutrition, and Metabolism = Physiologie Appliquee, Nutrition et Metabolisme, 34(5), 840–850. 10.1139/h09-088 19935845

[phy271107-bib-0016] Hughson, R. L. , Tschakovsky, M. E. , & Houston, M. E. (2001). Regulation of oxygen consumption at the onset of exercise. Exercise and Sport Sciences Reviews, 29(3), 129–133. 10.1097/00003677-200107000-00008 11474961

[phy271107-bib-0017] Jakovljevic, D. G. , Nunan, D. , Donovan, G. , Hodges, L. D. , Sandercock, G. , & Brodie, D. A. (2008). Comparison of cardiac output determined by different rebreathing methods at rest and at peak exercise. European Journal of Applied Physiology, 102, 593–599. 10.1007/s00421-007-0631-4 18074146

[phy271107-bib-0018] Johnson, S. T. , Tudor‐Locke, C. , McCargar, L. J. , & Bell, R. C. (2005). Measuring habitual walking speed of people with type 2 diabetes: Are they meeting recommendations? Diabetes Care, 28(6), 1503–1504. 10.2337/diacare.28.6.1503 15920080

[phy271107-bib-0019] Jones, A. M. , & Poole, D. C. (2005). Oxygen uptake dynamics: From muscle to mouth–an introduction to the symposium. Medicine and Science in Sports and Exercise, 37(9), 1542–1550. 10.1249/01.mss.0000177466.01232.7e 16177607

[phy271107-bib-0020] Keir, D. A. , Murias, J. M. , Paterson, D. H. , & Kowalchuk, J. M. (2014). Breath‐by‐breath pulmonary O2 uptake kinetics: Effect of data processing on confidence in estimating model parameters. Experimental Physiology, 99(11), 1511–1522. 10.1113/expphysiol.2014.080812 25063837

[phy271107-bib-0021] Kelley, D. E. , He, J. , Menshikova, E. V. , & Ritov, V. B. (2002). Dysfunction of mitochondria in human skeletal muscle in type 2 diabetes. Diabetes, 51(10), 2944–2950. 10.2337/diabetes.51.10.2944 12351431

[phy271107-bib-0022] Kiely, C. , O'Connor, E. , O'Shea, D. , Green, S. , & Egana, M. (2014). Hemodynamic responses during graded and constant‐load plantar flexion exercise in middle‐aged men and women with type 2 diabetes. Journal of Applied Physiology, 117, 755–764. 10.1152/japplphysiol.00555.2014 25123197

[phy271107-bib-0023] Kiely, C. , Rocha, J. , O'Connor, E. , O'Shea, D. , Green, S. , & Egana, M. (2015). Influence of menopause and type 2 diabetes on pulmonary oxygen uptake kinetics and peak exercise performance during cycling. American Journal of Physiology. Regulatory, Integrative and Comparative Physiology, 309(8), R875–R883. 10.1152/ajpregu.00258.2015 26269520

[phy271107-bib-0024] Mac Ananey, O. , Malone, J. , Warmington, S. , O'Shea, D. , Green, S. , & Egaña, M. (2011). Cardiac output is not related to the slowed o2 uptake kinetics in type 2 diabetes. Medicine and Science in Sports and Exercise, 43(6), 935–942. 10.1249/MSS.0b013e3182061cdb 21131874

[phy271107-bib-0025] Macananey, O. , O'Shea, D. , Warmington, S. A. , Green, S. , & Egaña, M. (2012). Gymnasium‐based unsupervised exercise maintains benefits in oxygen uptake kinetics obtained following supervised training in type 2 diabetes. Applied Physiology, Nutrition, and Metabolism, 37(4), 599–609. 10.1139/h2012-012 22563745

[phy271107-bib-0026] Madsen, S. M. , Thorup, A. C. , Overgaard, K. , Bjerre, M. , & Jeppesen, P. B. (2015). Functional and structural vascular adaptations following 8 weeks of low volume high intensity interval training in lower leg of type 2 diabetes patients and individuals at high risk of metabolic syndrome. Archives of Physiology and Biochemistry, 121(5), 178–186. 10.3109/13813455.2015.1087033 26471849

[phy271107-bib-0027] Morrato, E. H. , Hill, J. O. , Wyatt, H. R. , Ghushchyan, V. , & Sullivan, P. W. (2007). Physical activity in U.S. adults with diabetes and at risk for developing diabetes, 2003. Diabetes Care, 30(2), 203–209. 10.2337/dc06-1128 17259482

[phy271107-bib-0028] Mortensen, S. P. , Winding, K. M. , Iepsen, U. W. , Munch, G. W. , Marcussen, N. , Hellsten, Y. , Pedersen, B. K. , & Baum, O. (2019). The effect of two exercise modalities on skeletal muscle capillary ultrastructure in individuals with type 2 diabetes. Scandinavian Journal of Medicine & Science in Sports, 29(3), 360–368. 10.1111/sms.13348 30480353

[phy271107-bib-0029] O'Connor, E. , Green, S. , Kiely, C. , O'Shea, D. , & Egana, M. (2015). Differential effects of age and type 2 diabetes on dynamic vs. peak response of pulmonary oxygen uptake during exercise. Journal of Applied Physiology (Bethesda, MD: 1985), 118(8), 1031–1039. 10.1152/japplphysiol.01040.2014 25701005

[phy271107-bib-0030] O'Connor, E. , Kiely, C. , Gildea, N. , O'Shea, D. , Green, S. , & Egaña, M. (2025). Effects of pioglitazone with and without exercise training on cardiorespiratory fitness and oxygen uptake kinetics in type 2 diabetes. Diabetes, Obesity & Metabolism, 27(10), 5910–5920. 10.1111/dom.16648 PMC12409202 40735972

[phy271107-bib-0031] O'Connor, E. , Kiely, C. , O'Shea, D. , Green, S. , & Egaña, M. (2012). Similar level of impairment in exercise performance and oxygen uptake kinetics in middle‐aged men and women with type 2 diabetes. American Journal of Physiology. Regulatory, Integrative and Comparative Physiology, 303(1), R70–R76. 10.1152/ajpregu.00012.2012 22538515

[phy271107-bib-0032] Padilla, D. J. , McDonough, P. , Behnke, B. J. , Kano, Y. , Hageman, K. S. , Musch, T. I. , & Poole, D. C. (2006). Effects of type II diabetes on capillary hemodynamics in skeletal muscle. American Journal of Physiology. Heart and Circulatory Physiology, 291(5), H2439–H2444. 10.1152/ajpheart.00290.2006 16844923

[phy271107-bib-0033] Poitras, V. J. , Bentley, R. F. , Hopkins‐Rosseel, D. H. , LaHaye, S. A. , & Tschakovsky, M. E. (2015). Independent effect of type 2 diabetes beyond characteristic comorbidities and medications on immediate but not continued knee extensor hyperaemia. Journal of Applied Physiology, 119, 202–212. 10.1152/japplphysiol.00758.2014 PMC4526709 26048976

[phy271107-bib-0034] Poole, D. C. , & Jones, A. M. (2012). Oxygen uptake kinetics. Comprehensive Physiology, 2, 933–996. 10.1002/cphy.c100072 23798293

[phy271107-bib-0035] Regensteiner, J. G. , Bauer, T. A. , Reusch, J. E. , Brandenburg, S. L. , Sippel, J. M. , Vogelsong, A. M. , Smith, S. , Wolfel, E. E. , Eckel, R. H. , & Hiatt, W. R. (1998). Abnormal oxygen uptake kinetic responses in women with type II diabetes mellitus. Journal of Applied Physiology, 85(1), 310–317. 10.1152/jappl.1998.85.1.310 9655791

[phy271107-bib-0036] Rocha, J. , Gildea, N. , O'Shea, D. , Green, S. , & Egaña, M. (2019). Influence of priming exercise on oxygen uptake and muscle deoxygenation kinetics during moderate‐intensity cycling in type 2 diabetes. Journal of Applied Physiology (Bethesda, MD: 1985), 127(4), 1140–1149. 10.1152/japplphysiol.00344.2019 31414958

[phy271107-bib-0037] Ross, R. , Blair, S. N. , Arena, R. , Church, T. S. , Després, J. P. , Franklin, B. A. , Haskell, W. L. , Kaminsky, L. A. , Levine, B. D. , Lavie, C. J. , Myers, J. , Niebauer, J. , Sallis, R. , Sawada, S. S. , Sui, X. , & Wisløff, U. (2016). Importance of assessing cardiorespiratory fitness in clinical practice: A case for fitness as a clinical vital sign: A scientific Statement from the American Heart Association. Circulation, 134(24), e653–e699. 10.1161/cir.0000000000000461 27881567

[phy271107-bib-0038] Schreuder, T. H. , Green, D. J. , Nyakayiru, J. , Hopman, M. T. , & Thijssen, D. H. (2015). Time‐course of vascular adaptations during 8 weeks of exercise training in subjects with type 2 diabetes and middle‐aged controls. European Journal of Applied Physiology, 115(1), 187–196. 10.1007/s00421-014-3006-7 25260246

[phy271107-bib-0039] Soares, R. N. , & Lessard, S. J. (2024). Low response to aerobic training in metabolic disease: Role of skeletal muscle. Exercise and Sport Sciences Reviews, 52(2), 47–53. 10.1249/jes.0000000000000331 PMC10963145 38112622

[phy271107-bib-0040] Spencer, M. D. , Murias, J. M. , Lamb, H. P. , Kowalchuk, J. M. , & Paterson, D. H. (2011). Are the parameters of V̇o2, heart rate and muscle deoxygenation kinetics affected by serial moderate‐intensity exercise transitions in a single day? European Journal of Applied Physiology, 111, 591–600. 10.1007/s00421-010-1653-x 20931221

[phy271107-bib-0041] Wei, M. , Gibbons, L. W. , Kampert, J. B. , Nichaman, M. Z. , & Blair, S. N. (2000). Low cardiorespiratory fitness and physical inactivity as predictors of mortality in men with type 2 diabetes. Annals of Internal Medicine, 132(8), 605–611. 10.7326/0003-4819-132-8-200004180-00002 10766678

[phy271107-bib-0042] Wilkerson, D. P. , Poole, D. C. , Jones, A. M. , Fulford, J. , Mawson, D. M. , Ball, C. I. , & Shore, A. C. (2011). Older type 2 diabetic males do not exhibit abnormal pulmonary oxygen uptake and muscle oxygen utilization dynamics during submaximal cycling exercise. American Journal of Physiology. Regulatory, Integrative and Comparative Physiology, 300(3), R685–R692. 10.1152/ajpregu.00479.2010 21178129

[phy271107-bib-0043] Wilson, G. A. , Wilkins, G. T. , Cotter, J. D. , Lamberts, R. R. , Lal, S. , & Baldi, J. C. (2017). Impaired ventricular filling limits cardiac reserve during submaximal exercise in people with type 2 diabetes. Cardiovascular Diabetology, 16(160), 1–8. 10.1186/s12933-017-0644-1 PMC5735887 29258502

[phy271107-bib-0044] Wilson, G. A. , Wilkins, G. T. , Cotter, J. D. , Lamberts, R. R. , Lal, S. , & Baldi, J. C. (2019). HIIT improves left ventricular exercise response in adults with type 2 diabetes. Medicine and Science in Sports and Exercise, 51, 1099–1105. 10.1249/mss.0000000000001897 30640284

[phy271107-bib-0045] Wilson, G. A. , Wilson, L. C. , Lamberts, R. R. , Majeed, K. , Lal, S. , & Baldi, J. C. (2017). Beta‐adrenergic responsiveness in the type 2 diabetic heart: Effects on cardiac reserve. Medicine and Science in Sports and Exercise, 49(5), 907–914. 10.1249/MSS.0000000000001184 27984428

[phy271107-bib-0046] Winding, K. M. , Munch, G. W. , Iepsen, U. W. , Van Hall, G. , Pedersen, B. K. , & Mortensen, S. P. (2018). The effect on glycaemic control of low‐volume high‐intensity interval training versus endurance training in individuals with type 2 diabetes. Diabetes, Obesity & Metabolism, 20(5), 1131–1139. 10.1111/dom.13198 29272072

